# Preterm delivery and small-for-gestation outcomes in HIV-infected pregnant women on antiretroviral therapy in rural South Africa: Results from a cohort study, 2010-2015

**DOI:** 10.1371/journal.pone.0192805

**Published:** 2018-02-22

**Authors:** Terusha Chetty, Claire Thorne, Anna Coutsoudis

**Affiliations:** 1 Africa Health Research Institute, Mtubatuba, KwaZulu-Natal, South Africa; 2 Department of Public Health Medicine, School of Nursing and Public Health, University of KwaZulu-Natal, Durban, KwaZulu-Natal, South Africa; 3 UCL Great Ormond Street Institute of Child Health, University College London, London, United Kingdom; 4 Department of Paediatrics and Child Health, School of Clinical Medicine, University of KwaZulu-Natal, Durban, KwaZulu-Natal, South Africa; Katholieke Universiteit Leuven Rega Institute for Medical Research, BELGIUM

## Abstract

**Objectives:**

Increasingly more women conceive on antiretroviral therapy (ART) with non-nucleoside reverse transcriptase-based regimens. This study assessed the effect of preconception tenofovir disoproxil fumarate (TDF)-lamivudine (3TC)/emtricitabine (FTC)-efavirenz (EFV) and post-conception TDF-(3TC/FTC)-EFV (versus other regimens) on preterm delivery (PTD) and small-for-gestational age (SGA) births.

**Methods:**

We analysed data of 2549 HIV-infected women attending antenatal clinics in KwaZulu-Natal from 2010 through 2015 in this retrospective cohort study. Preconception, TDF-(3TC/FTC)-EFV was compared to nevirapine (NVP)-based regimens and other 3-drug EFV-based regimens. Post-conception, TDF-(3TC/FTC)-EFV was compared to NVP-based ART and zidovudine (ZDV) prophylaxis. Outcomes included PTD <37 weeks and SGA births. Generalized linear mixed effects were used to fit logistic regression models to account for repeat pregnancies.

**Results:**

Among 2549 singleton live births, 10.4% (n = 264) were PTD and 10.4% (n = 265) SGA. PTD declined from 16.3% in 2010 to 9.3% in 2015 and SGA remained stable from 9.9% in 2010 to 10% in 2015.

Preconception NVP-based regimens [adjusted odds ratio (aOR) 0.66; 95% CI 0.27–1.63] and other 3-drug EFV-based regimens (aOR 0.72; 95% CI 0.24–2.12) were not associated with PTD versus TDF-(3TC/FTC)-EFV. NVP-based (aOR 0.75; 95% CI 0.40–1.42) and other 3-drug EFV-based regimens (aOR 1.55; 95% CI 0.76–3.16) were not associated with SGA births versus TDF-(3TC/FTC)-EFV.

Post-conception NVP-based ART (1.77; 95% CI 0.89–3.51) and ZDV (1.03; 95% CI 0.68–1.58) were not associated with PTD versus TDF-(3TC/FTC)-EFV. NVP-based ART (1.55; 95% CI 0.66–3.61) and ZDV (0.89; 95% CI 0.53–1.47) were not associated with SGA versus TDF-(3TC/FTC)-EFV.

**Conclusions:**

Preconception TDF-(3TC/FTC)-EFV and post-conception TDF-(3TC/FTC)-EFV were not associated with PTD or SGA, compared with other regimens. Increasing ART use merits further study of the optimum ART regimen for safe birth outcomes.

## Introduction

Triple antiretroviral therapy (ART) reduces perinatal HIV transmission [1–3] and improves maternal and infant survival [4,5]. However, ART may increase adverse birth outcomes, including preterm delivery (PTD) and small-for-gestational age (SGA) births in developed [6,7] and developing settings [8–11]. Until recently, birth data for ART-exposed pregnancies mostly came from cohorts in developed countries [6,12–16], frequently with small sample size [7,17,18] and with more information on protease inhibitor (PI)-based regimens then non-nucleoside reverse transcriptase inhibitors (NNRTI) [8,19].

In the Promoting Maternal and Infant Survival Everywhere (PROMISE) trial, zidovudine (ZDV)-based ART [ZDV, lamivudine (3TC) and lopinavir-ritonavir (LPV/r)] and tenofovir-based ART [tenofovir disoproxil fumarate (TDF), emtricitabine (FTC) and LPV/r] increased PTD risk versus ZDV alone; TDF-based ART significantly increased PTD risk before 34 weeks versus ZDV-based ART [8]. In a large Botswana cohort, TDF-FTC-efavirenz (EFV) regimens were observed to be at least as safe as other regimens and reduced SGA risk [20]. These findings were confirmed in a Botswana surveillance study where preconception TDF-FTC-EFV had lower risk for any adverse birth outcome and protected against severe adverse birth outcomes versus TDF-FTC-nevirapine (NVP) or ZDV-3TC-NVP [21]. Relative to TDF-FTC-EFV, all regimens increased SGA risk and ZDV-3TC-NVP increased very PTD delivery risk [21].

South African guidelines for women initiating post-conception ART evolved from dual therapy with ZDV and NVP (Option A) in 2010 to interrupted ART with TDF-FTC-EFV (Option B) in 2013 and lifelong ART with TDF-FTC-EFV (Option B+) in 2015 [22–24]. Globally, South Africa has the largest absolute number of people on ART (3,929,000), with almost 56% of HIV-infected people on ART in 2016 [25,26]. Over 95% of HIV-infected pregnant women receive ART for prevention of mother-to-child transmission (PMTCT) [27]. As more women conceive on ART [27–30], PTD and SGA births may become more evident. Our objective was to determine the association between preconception TDF-(3TC/FTC)-EFV and post-conception TDF-(3TC/FTC)-EFV and risk of PTD and SGA births, versus other regimens. A secondary objective was to present annual trends in PTD and SGA births.

## Materials and methods

Between 2010 and 2012, HIV-infected pregnant women attending any one of the 17 antenatal clinics in the Hlabisa HIV Treatment and Care Programme, northern KwaZulu-Natal, South Africa were eligible for inclusion [31]. Thereafter enrolment was limited to seven clinics within the Africa Centre surveillance area which allowed data linkage (clinics were from the original cohort with 40% of patients generally representative of Hlabisa) [32]; the additional clinic (Mtubatuba) was one of the busiest in the sub-district [33].

Clinic staff collected antenatal data [31]. Birth outcomes were abstracted from hospital and clinic records. Antenatal data were linked with HIV clinical data [CD4^+^, viral load (VL), ART regimen, initiation dates, and prior PMTCT] [33].

Singleton live births of at least 24 weeks to HIV-infected women with a recorded delivery birthweight and gestational age between 1 January 2010 and 31 December 2015 were included. We excluded multiple births given the increased LBW and PTD risk.

### ART groups, regimen and timing

Dates of ART initiation or change were retrieved from the HIV clinical database [33]. We categorised pregnancies as starting ART before pregnancy (“preconception ART”) or “post-conception ARV” (pregnancy-initiated ART or prophylaxis). Preconception ART were classified into: TDF-(3TC/FTC)-EFV; NVP-based regimens; and other 3-drug EFV-based regimens. Post-conception ARV were categorized into: TDF-(3TC/FTC)-EFV, NVP-based ART and ZDV prophylaxis. Preconception, TDF-(3TC/FTC-EFV), other 3-drug EFV-based regimens [ABC-3TC-EFV; ZDV-3TC-EFV; stavudine (d4T)-3TC-EFV], and NVP-based regimens (TDF-3TC-NVP; ZDV-3TC-NVP; d4T-3TC-NVP), and post-conception TDF-(3TC/FTC)-EFV and NVP-based regimens (ZDV-3TC-NVP, TDF-3TC-NVP, d4T-3TC-NVP) were combined as there were no differences in adverse outcomes between individual regimens in crude and adjusted analyses.

Preconception ART initiation timing was calculated from initiation date to last menstrual period (LMP). Post-conception ARV initiation was categorized into first, second, or third trimester.

### Definitions

Nurses determined gestational age at the first antenatal visit, using LMP, symphysis-fundal height, or ultrasound (for hospital referrals). In women without antenatal care (under 0.5% in both groups), gestational age was estimated by LMP history and infant birth examination. Surveillance birth data were also used to verify gestational age where available. Gestational age was recorded on the maternal delivery record.

SGA was defined as birthweight below 10th percentile of expected weight for gestational age [34,35]. Birthweights at or above 10^th^ percentile were appropriate for gestational age (AGA). Low birth weight (LBW) was birthweight below 2.5 kg. PTD were births before 37 completed pregnancy weeks.

Antenatal clinic type was classified as rural, peri-urban or other (care outside Hlabisa) [36]. Parity data was not collected. As some women experienced additional pregnancies between 2010 and 2015, pregnancies were classified as index or repeat, then categorized as first, second or third pregnancy (“pregnancy count”). Delivery mode was classified as caesarean section or vaginal delivery. The last pregnancy CD4^+^ was used in analyses; for women without post-conception CD4^+^ measurement, the measure closest to delivery through six weeks postpartum was used. As pregnancy VLs was not recommended until 2013, only women on preconception ART had post-conception VLs.

### Statistical analysis

Logistic regression models were used to evaluate PTD and SGA risk factors. We ran separate models by ART initiation timing (preconception or post-conception). Pregnancies with regimen switches or on PI-based ART were excluded. Post-conception, we restricted analyses to women with at least four weeks of antiretroviral exposure to limit lead time bias. To allow for repeat pregnancies, generalized linear mixed effects accounted for maternal random effects.

Variables considered a priori to be important, maternal age at first post-conception visit (15–24 years, 25–34 years; ≥35 years), ART timing, CD4^+^ (0–100, 101–200, 200–350, >350, or missing) and calendar birth year, were retained in multivariable models [11]. Other covariates including antenatal clinic type, delivery place, infant sex, and pregnancy count were included in univariable analyses as potential confounding factors and included in multivariable models if *P* value <0.2. As the cohort was designed to determine PMTCT programme effectiveness, other confounding factors such as diabetes, hypertension, smoking, drug abuse, prior PTD history, body mass index, food insecurity and other infections were not collected. Interaction terms were included to determine if age, calendar birth year, or ART timing modified the regimen and birth outcomes association, but was not included in the final models as no association was found.

We needed 3591 women to show 2% difference in PTD between preconception groups and 1186 women to show 3.5% PTD difference in post-conception groups with 80% power at the 0.05 confidence level. Using the current sample size of 968 in the preconception group and 1581 in the post-conception group, the study was powered at under 20% and 30%, respectively. Two-sided *P* values <0.05 were considered statistically significant. Analyses were conducted using Stata 13.1.

### Sensitivity analysis

We performed several sensitivity analyses to address issues in population, exposure and outcome definitions.

To address historical bias, we restricted the analysis to the seven surveillance clinics from 2010–2015 (analysis 1). In a second sensitivity analysis, we excluded deliveries in 2010 and 2011 when birth outcome rates were higher. In analysis 3, we excluded calendar year as an explanatory variable as this may have been too closely associated with HIV guideline changes. We also assessed the seven clinics from 2012–2015 to determine whether changes in risk estimates were related to calendar year. We included LBW as an outcome to address the uncertainty around gestational age dating (analysis 4). To address lead time bias, we restricted the post-conception analysis to those who started ART under 24 weeks allowing sufficient time for PTD (analysis 5).

### Ethics approval and consent to participate

The University of KwaZulu-Natal Biomedical Research Ethics Committee granted ethics approval for retrospective analysis of routine data collected in Hlabisa and Mtubatuba local municipalities health centres (BE066/07) and for this analysis (BE002/16). As patient movement within antenatal clinics was dynamic with different entry times, including delivery, reliable written informed consent was challenging. We therefore requested a waiver of written informed consent for pregnancy data linkage (E134/06). Instead, women gave verbal consent for data linkage.

## Results

**Fig 1:**
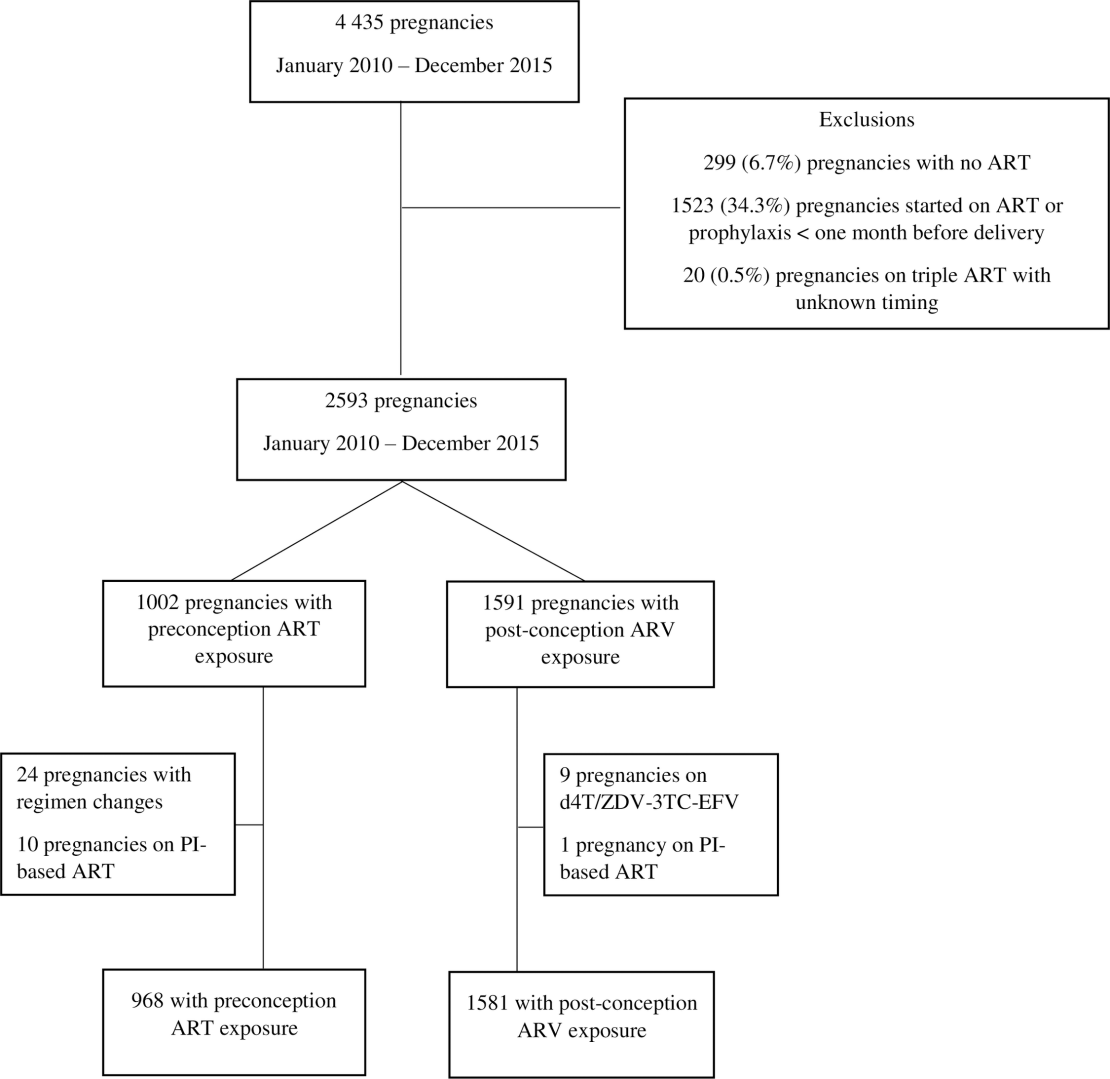
Study inclusions and exclusions.

Of 4435 pregnancies in 4161 HIV-infected women, 299 (6.7%) were unexposed to ART, 1523 (34.3%) had under one month ARV exposure, and 20 (0.5%) had unknown ART timing (Fig 1). Of 2593 pregnancies remaining, 1002 initiate preconception ART and 1591 started post-conception ARV. Most of the post-conception group were on ZDV-prophylaxis or NNRTI-based regimens; we excluded nine pregnancies on d4T/ZDV-3TC-EFV as there were no adverse outcomes and one pregnancy with PI-based ART. Ten pregnancies on PI-based ART and 24 with pregnancy regimen changes were excluded from the preconception group, leaving 2549 pregnancies for this analysis; 968 (38.0%) were on preconception ART and 1581 (62.0%) on post-conception ARV.

### Population characteristics

In the preconception group, TDF-(3TC/FTC)-EFV was the predominant regimen (532, 55.0%), 95 of which were TDF-FTC-EFV (17.9%) (Table 1). In the post-conception group, TDF-(3TC/FTC)-EFV was the predominant regimen (959, 60.7%), 667 of which were TDF-FTC-EFV (69.6%), 5.9% on NVP-based regimens and 33.4% on ZDV prophylaxis. The post-conception group were younger than the preconception group. Late third trimester antenatal attendance was common across most groups except post-conception TDF-(3TC/FTC)-EFV and ZDV. Most women attended rural antenatal clinics and had vaginal deliveries in hospital (Tables 1 and 2). Overall, women were relatively healthy with median CD4^+^ count above 350 cells/mm^3^, except women on NVP-based (post-conception), consistent with earlier guidelines and CD4^+^ cutoff (Table 1). Preconception, the median VL was ≤ 40 copies/ml (undetectable) in all ART groups. However, among 616 women with VLs, 75 (12.2%) had VL above 1000 copies/ml (Table 1); 9.3% of pregnancies had VL above 1000 copies/ml in TDF-(3TC/FTC)-EFV groups (30/324) and 15.4% (45/292) in other 3-drug EFV and NVP-based groups. Preconception, median ART duration was 91 weeks (interquartile range (IQR) 51 weeks-147 weeks), with women on TDF-(3TC/FTC)-EFV having the shortest duration. Post-conception groups usually initiated treatment in the second trimester, but 11.5% (182/1581) overall initiated in the first trimester.

**Table 1:** Maternal, pregnancy and delivery characteristics by antiretroviral regimen.

Characteristics	Preconception ART group (N = 968)			Post-conception ARV group (N = 1581)		
	TDF-(3TC/FTC)-EFV ^a^	NVP-based regimen ^b^	Other 3-drug EFV-based regimen ^c^	TDF-(3TC/FTC)-EFV initiated in pregnancy ^d^	NVP-based ART initiated in pregnancy ^e^	ZDV initiated in pregnancy
	(n = 532)	(n = 249)	(n = 187)	(n = 959)	(n = 94)	(n = 528)
	n (%)	n (%)	n (%)	n (%)	n (%)	n (%)
**Maternal age, years**						
Median (IQR)	30 (26–34)	30 (26–33)	32 (28–36)	27 (23–31)	29 (23–34)	25 (21–30)
15–24	102 (19.2)	37 (14.9)	18 (9.6)	345 (36.0)	30 (31.9)	263 (49.8)
25–34	323 (60.7)	159 (63.9)	101 (54.0)	492 (51.3)	43 (45.7)	223 (42.2)
≥35	107 (20.1)	53 (21.3)	68 (36.4)	122 (12.7)	21 (22.3)	42 (8.0)
**Gestation at first antenatal visit, weeks**						
1–12	57 (10.7)	19 (7.6)	14 (7.5)	125 (13.0)	3 (3.2)	54 (10.2)
13–26	219 (41.2)	79 (31.7)	48 (25.7)	541 (56.4)	31 (33.0)	337 (63.8)
27–44	256 (48.1)	151 (60.6)	125 (66.8)	293 (30.6)	60 (63.8)	137 (26.0)
**Enrolment clinic**						
Rural	301 (56.6)	167 (67.1)	134 (71.7)	434 (45.3)	67 (71.3)	334 (63.3)
Peri-urban	223 (41.9)	74 (29.7)	49 (26.2)	519 (54.1)	26 (27.7)	192 (36.4)
Other	7 (1.3)	6 (2.4)	3 (1.6)	4 (0.4)	-	-
Missing	1 (0.2)	2 (0.8)	1 (0.5)	2 (0.2)	1 (1.1)	2 (0.4)
**CD4**^**+**^ **count, cells/mm**^**3**^						
Median (IQR)	494 (328–685)	426 (299–575)	423 (296–593)	437 (285–639)	302 (221–433)	443 (337–601)
0–100	10 (1.9)	6 (2.4)	3 (1.6)	20 (2.1)	6 (6.4)	11 (2.1)
101–200	29 (5.5)	21 (8.4)	12 (6.4)	74 (7.7)	14 (14.9)	31 (5.9)
201–350	77 (14.5)	42 (16.9)	38 (20.3)	233 (24.3)	39 (41.5)	79 (15.0)
>350	295 (55.5)	139 (55.8)	102 (54.6)	517 (53.9)	33 (35.1)	320 (60.6)
Missing	121 (22.7)	41 (16.5)	32 (17.1)	115 (12.0)	2 (2.1)	87 (16.5)
Viral load, copies/ml*						
≤1000	294 (55.3)	144 (57.8)	103 (55.1)			
>1000	30 (5.6)	24 (9.6)	21 (11.2)			
ART initiation timing**						
Median (IQR), weeks	74 (41–118)	99 (56–142)	148 (86–239)			
1st trimester				114 (11.9)	6 (6.4)	62 (11.7)
2nd trimester				563 (58.7)	45 (47.9)	331 (62.7)
3rd trimester				282 (29.4)	43 (45.7)	135 (25.6)

^a^ TDF-3TC-EFV (82.1%); and TDF-FTC-EFV (17.9%)

^b^ ZDV-3TC-NVP (4.8%); d4T-3TC-NVP (57.8%); TDF-3TC-NVP (37.3%)

^c^ ABC-3TC-EFV (1.1%); ZDV-3TC-EFV (8.0%); d4T-3TC-EFV (90.9%)

^d^ TDF-3TC-EFV (30.5%); TDF-FTC-EFV (69.6%)

^e^ ZDV-3TC-NVP (21.3%); d4T-3TC-NVP (7.5%); TDF-3TC-NVP (71.3%)

*Insufficient data was available for the pregnancy ARV group

**Preconception ART timing was calculated from the date of initiation to the LMP

**Table 2:** Delivery characteristics by antiretroviral regimen.

Characteristics	Preconception ART group (N = 968)			Post-conception ARV group (N = 1581)		
	TDF-(3TC/FTC)-EFV ^a^	NVP-based regimen ^b^	Other 3-drug EFV-based regimen ^c^	TDF-(3TC/FTC)-EFV initiated in pregnancy ^d^	NVP-based ART initiated in pregnancy ^e^	ZDV initiated in pregnancy
	(n = 532)	(n = 249)	(n = 187)	(n = 959)	(n = 94)	(n = 528)
	n (%)	n (%)	n (%)	n (%)	n (%)	n (%)
**Type of delivery**						
Caesarean	58 (10.9)	22 (8.8)	13 (7.0)	95 (9.9)	3 (3.2)	46 (8.7)
Vaginal	439 (82.5)	137 (55.0)	88 (47.1)	689 (71.9)	21 (22.3)	241 (45.6)
Missing	35 (6.6)	90 (36.1)	86 (46.0)	175 (18.3)	70 (74.5)	241 (45.6)
**Gestational age at delivery, weeks**						
Median (IQR)	39 (38–40)	38 (38–40)	39 (38–40)	39 (38–40)	39 (37–40)	39 (38–40)
<32	3 (0.6)	2 (0.8)	1 (0.5)	10 (1.0)	-	3 (0.6)
32–33	5 (0.9)	1 (0.4)	1 (0.5)	14 (1.5)	2 (2.1)	6 (1.1)
34–36	41 (7.7)	21 (8.4)	19 (10.2)	82 (8.5)	12 (12.8)	41 (7.8)
≥37	483 (90.8)	225 (90.4)	166 (88.8)	853 (89.0)	80 (85.1)	478 (90.5)
**Preterm delivery**						
No	483 (90.8)	225 (90.4)	166 (88.8)	853 (89.0)	80 (85.1)	478 (90.5)
Yes	49 (9.2)	24 (9.6)	21 (11.2)	106 (11.0)	14 (14.9)	50 (9.5)
**Birthweight (kg)**						
Median (IQR)	3.1 (2.8–3.4)	3.1 (2.8–3.4)	3.1 (2.7–3.4)	3.1 (2.8–3.4)	2.9 (2.7–3.2)	3.1 (2.8–3.4)
≥2.5	480 (90.2)	231 (92.8)	171 (91.4)	856 (89.3)	84 (89.4)	482 (91.3)
<2.5	52 (9.8)	18 (7.2)	16 (8.6)	103 (10.7)	10 (10.6)	46 (8.7)
**Size for gestation**						
AGA	475 (89.3)	230 (92.4)	163 (87.2)	864 (90.1)	80 (85.1)	472 (89.4)
SGA	57 (10.7)	19 (7.6)	24 (12.8)	95 (9.9)	14 (14.9)	56 (10.6)
**Calendar year of delivery**						
2010	-	27 (10.8)	18 (9.6)	58 (6.1)	20 (21.3)	18 (3.4)
2011	14 (2.6)	52 (20.9)	61 (32.6)	108 (11.3)	38 (40.4)	196 (37.1)
2012	51 (9.6)	39 (15.7)	30 (16.0)	70 (7.3)	34 (36.2)	124 (23.5)
2013	106 (19.9)	46 (18.5)	19 (10.2)	141 (14.7)	2 (2.1)	115 (21.8)
2014	216 (40.6)	54 (21.7)	42 (22.5)	350 (36.5)	-	49 (9.3)
2015	145 (27.3)	31 (12.5)	17 (9.1)	232 (24.2)	-	26 (4.9)

^a^ TDF-3TC-EFV (82.1%); and TDF-FTC-EFV (17.9%)

^b^ ZDV-3TC-NVP (4.8%); d4T-3TC-NVP (57.8%); TDF-3TC-NVP (37.3%)

^c^ ABC-3TC-EFV (1.1%); ZDV-3TC-EFV (8.0%); d4T-3TC-EFV (90.9%)

^d^ TDF-3TC-EFV (30.5%); TDF-FTC-EFV (69.6%)

^e^ ZDV-3TC-NVP (21.3%); d4T-3TC-NVP (7.5%); TDF-3TC-NVP (71.3%)

Post-conception, PTD by ART start were: 8.2% (15/182) in the first, 9.8% (92/939) in the second and 13.7% (63/460) in the third trimester. SGA by ART initiation were: 11.5% (21/182) in the first, 9.9% (93/939) in the second and 11.1% (51/460) in the third trimester.

### Preterm delivery and SGA trends over time

**Fig 2:**
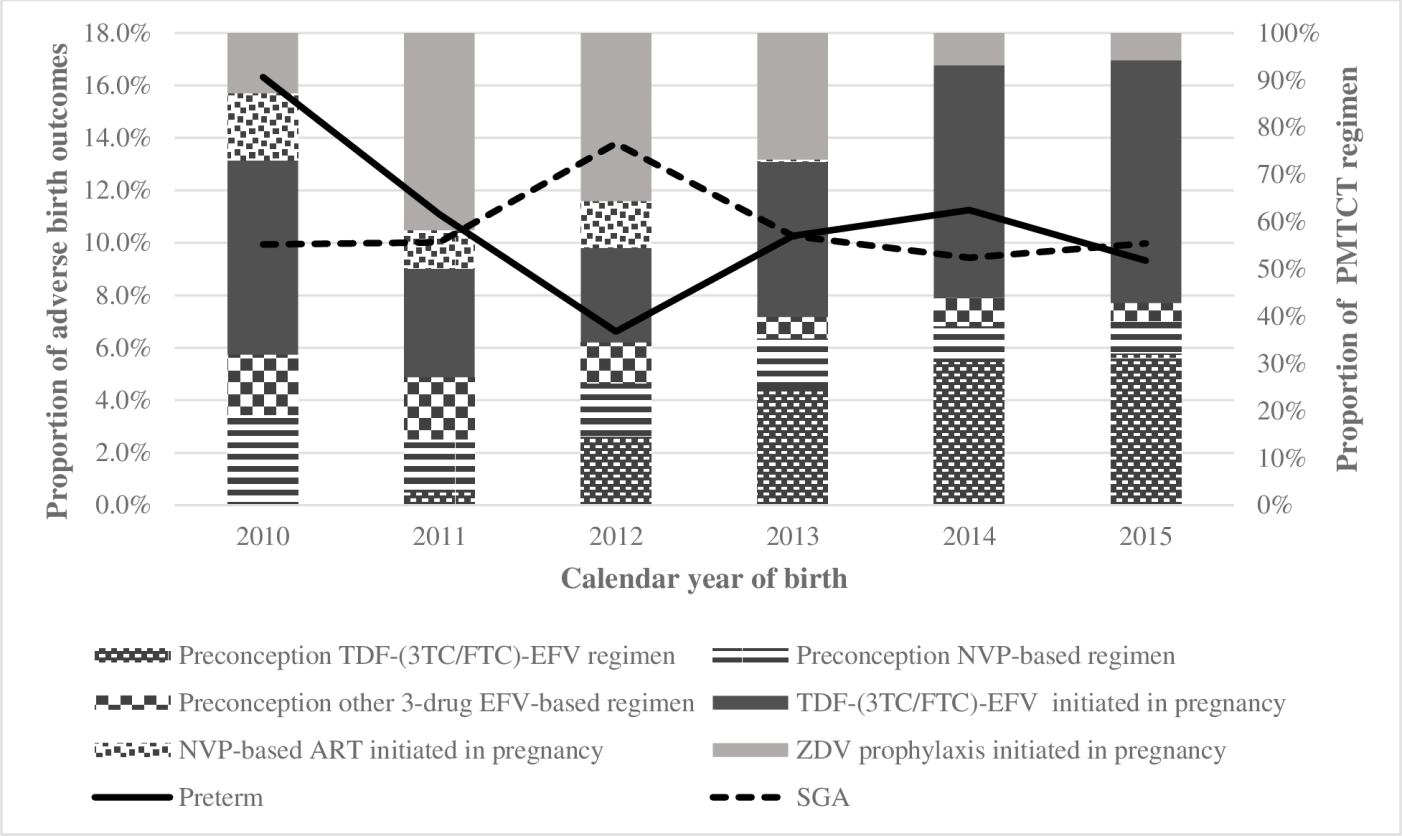
PTD and SGA trends by year and PMTCT regimen.

Fig 2 shows evolving ART over time, with increased preconception TDF-(3TC/FTC)-EFV (2.6% in 2011 to 27.3% in 2015) and post-conception EFV-based ART (12% in 2011 to 25.7% in 2015). No one received preconception TDF-(3TC/FTC)-EFV or post-conception TDF/FTC/EFV in 2010. Post-conception TDF-3TC-EFV declined in 2012 stopping after 2014; concurrently TDF-FTC-EFV increased from 2012 aligned with guidelines [24,37]. NVP-based ART stopped in 2014.

Overall, there were 245 LBW (9.6%) infants, 264 PTDs (10.4%) and 265 SGA infants (10.4%). Nineteen PTD infants (7.2%) were also SGA. PTDs fluctuated from 16.3% (23/141) in 2010, 6.6% (23/348) in 2012, to 9.3% in 2015 (42/451). SGA births were approximately 10% through the years, peaking at 13.8% (48/348) in 2012 (Fig 2).

Preconception, PTD declined from 20.0% in 2010 (9/45) to 7.7% in 2015 (15/193). PTD was ~7% in TDF-(3TC/FTC)-EFV in 2011 (1/14), 2013 (8/106) and 2015, highest in 2014 (11.1%; 24/216). In 2015, PTDs were highest in NVP-based ART and other 3-drug EFV-based ART (11.8%; 2/17) and lowest in TDF-(3TC/FTC)-EFV ART (7.6%; 11/145). NVP-based ART had the highest PTDs in 2010 and 2011 (50%; 1/2 and 7/14 respectively).

Preconception, SGAs increased from 6.7% (3/45) in 2010 to 11.7% (23/196) in 2015. SGA increased from 7.1% (1/14) in TDF-(3TC/FTC)-EFV in 2011 to 13.1% (19/145) in 2015. The 2012 sharp SGA incline was driven by preconception ART: NVP-based (64.3%; 9/14) and other 3-drug EFV-based ART (10/30; 33.3%).

Post-conception, PTD declined from 14.6% in 2010 (14/96) to 10.5% in 2015 (27/258) with nadir of 5.7% in 2012 (13/228). PTD in TDF-(3TC/FTC)-EFV was highest in 2011 (13.0%; 14/108) and declined to 11.2% in 2015 (26/232).

Post-conception, SGAs dropped from 11.5% (11/96) in 2010 to 8.5% in 2015 (22/258). SGAs declined from 10.3% in 2010 (6/58) to 8.2% in 2015 (19/232) in TDF-(3TC/FTC)-EFV and from 16.7% (3/18) in 2010 to 11.5% (3/26) in 2015 when on ZDV.

### Birth outcomes by preconception ART (N = 968)

Preconception regimens were not associated with PTD and SGA in univariable and multivariable analyses (Table 3). In the PTD univariable analysis, the adjusted odds ratio (aOR) for NVP-based and other 3-drug EFV-based regimens was 1.10 (95% CI 0.52–2.31) and 1.35 (95% CI 0.60–3.05) respectively, compared with TDF-(3TC/FTC)-EFV (Table 3).

**Table 3:** Risk factors for PTD and SGA deliveries between 2010 and 2015 in women on preconception ART (N = 968).

		Preterm				SGA			
Characteristic	n (%)	Unadjusted OR (95% CI)	*P*	Adjusted OR (95% CI)	*P*	Unadjusted OR (95% CI)	*P*	Adjusted OR (95% CI)	*P*
**ART regimen**	**968**								
TDF-(3TC/FTC)-EFV	532 (54.96)	Ref	Ref			Ref		Ref	-
NVP-based regimen	249 (25.72)	1.10 (0.52–2.31)	0.807	0.66 (0.27–1.63)	0.366	0.69 (0.40–1.18)	0.177	0.75 (0.40–1.42)	0.380
Other 3-drug EFV-based regimen	187 (19.32)	1.35 (0.60–3.05)	0.465	0.72 (0.24–2.12)	0.550	1.23 (0.74–2.04)	0.431	1.55 (0.76–3.16)	0.226
Time on ART, weeks*	968 (100)	1.00 (0.99–1.00)	0.823	1.00 (0.99–1.01)	0.290	0.99 (0.99–1.00)	0.687	1.00 (0.99–1.00)	0.689
**CD4**^**+**^ **category, cells/mm**^**3**^	**968**								
0–100	19 (1.96)	Ref	-	Ref	-	Ref	-	Ref	-
101–200	62 (6.40)	1.11 (0.10–14.3)	0.931	1.31 (0.13–13.66)	0.820	0.77 (0.18–3.21)	0.332	0.49 (0.11–2.24)	0.356
200–350	157 (16.22)	0.70 (0.10–7.65)	0.771	0.83 (0.09–7.49)	0.866	0.91 (0.25–3.36)	0.647	0.66 (0.17–2.54)	0.542
>350	536 (55.37)	0.83 (0.10–8.10)	0.869	0.98 (0.12–7.96)	0.981	0.68 (0.19–2.39)	0.407	0.58 (0.16–2.10)	0.405
Missing	194 (20.04)	0.95 (0.10–9.84)	0.963	1.19 (0.14–10.37)	0.874	0.43 (0.11–1.64)	0.133	0.35 (0.09–1.39)	0.137
**Age, years**	**968**								
25–34	583 (60.23)	Ref	-	Ref	-	Ref	-	Ref	-
15–24	157 (16.22)	1.73 (0.73–4.09)	0.210	1.72 (0.73–4.01)	0.213	1.33 (0.78–2.25)	0.296	1.28 (0.73–2.27)	0.389
≥ 35	228 (23.55)	1.07 (0.49–2.32)	0.874	0.98 (0.46–2.07)	0.945	1.05 (0.64–1.72)	0.832	1.03 (0.61–1.76)	0.905
**Calendar year of delivery**	**968**								
2010	45 (4.65)	Ref	-	Ref	-	Ref	-	Ref	-
2011	127 (13.12)	0.40 (0.10–1.66)	0.208	0.34 (0.08–1.51)	0.156	1.11 (0.29–4.23)	0.877	0.79 (0.19–3.23)	0.740
2012	120 (12.40)	0.25 (0.10–1.13)	0.072	0.19 (0.04–0.98)	0.047	3.92 (1.13–13.6)	0.031	4.16 (1.14–15.07)	0.030
2013	171 (17.67)	0.33 (0.10–1.31)	0.116	0.24 (0.05–1.13)	0.072	1.51 (0.42–5.38)	0.528	1.62 (0.42–6.24)	0.483
2014	312 (32.23)	0.28 (0.10–1.10)	0.066	0.17 (0.03–0.93)	0.041	1.23 (0.36–4.24)	0.743	1.40 (0.36–5.41)	0.629
2015	193 (19.94)	0.23 (0.06–0.98)	0.047	0.13 (0.02–0.82)	0.030	1.86 (0.53–6.49)	0.330	2.30 (0.56–9.34)	0.245

*Timing of ART in the preconception groups is calculated from the date of initiation to the last menstrual period

Antenatal clinic type, VL, pregnancy count, delivery place and infant sex were excluded from the model as *P* >0.2

Multivariably, adjusting for ART duration, CD4^+^ count, age, and calendar delivery year, NVP-based (aOR 0.66; 95% CI 0.27–1.63) and other 3-drug EFV-based regimens (aOR 0.72; 95% CI 0.24–2.12) non-significantly reduced PTD risk versus TDF-(3TC/FTC)-EFV (Table 3).

In univariable analysis, NVP-based regimens non-significantly lowered SGA risk to 0.69 (95% CI 0.40–1.18); other 3-drug EFV-based regimens increased SGA risk to 1.23 (95% CI 0.74–2.04) versus TDF-(3TC/FTC)-EFV (Table 3). Multivariably, NVP-based regimens (aOR 0.75; 95% CI 0.40–1.42) non-significantly reduced SGA risk; the aOR for other 3-drug EFV-based regimens was 1.55 (95% CI 0.76–3.16) versus TDF-(3TC/FTC)-EFV (Table 3).

In sensitivity analyses (S1 Table), PTD and SGA risk in other regimens versus TDF-(3TC/FTC)-EFV were not substantially altered by restricting clinics and calendar year or excluding calendar year (SGA estimates were more precise). When LBW was an outcome, risk for NVP-based regimens and other 3-drug EFV-based regimens changed to 0.76 and 1.28 (analysis 4), respectively, with wider confidence intervals than the main PTD analysis.

### Birth outcomes by post-conception ARV (N = 1581)

Post-conception ARV was not associated with PTD and SGA in crude and adjusted models (Table 4). In the univariable analysis, PTD risk was 1.41 (95% CI 0.77–2.57) for NVP-based ART and 0.84 (95% CI 0.59–1.20) for ZDV prophylaxis versus TDF-(3TC/FTC)-EFV (Table 4).

**Table 4:** Risk factors for preterm and SGA deliveries between 2010 and 2015 in women initiating post-conception ARV (n = 1581).

		Preterm				SGA			
Characteristic	n (%)	Unadjusted OR (95% CI)	*P*	Adjusted OR (95% CI)	*P*	Unadjusted OR (95% CI)	*P*	Adjusted OR (95% CI)	*P*
**ART regimen**	**1581**								
TDF-(3TC/FTC)-EFV	959 (42.19)	Ref	Ref	Ref		Ref		Ref	
NVP-based ART	94 (5.95)	1.41 (0.77–2.57)	0.266	1.77 (0.89–3.51)	0.104	1.74 (0.79–3.86)	0.171	1.55 (0.66–3.61)	0.311
ZDV prophylaxis	528 (33.40)	0.84 (0.59–1.20)	0.341	1.03 (0.68–1.58)	0.878	1.09 (0.72–1.65)	0.675	0.89 (0.53–1.47)	0.639
**Timing of ART initiation**	**1581**								
Second trimester	939 (59.39)	Ref	-	Ref	-	Ref	-	Ref	
First trimester	182 (11.51)	0.83 (0.47–1.46)	0.514	0.77 (0.43–1.38)	0.380	1.24 (0.67–2.31)	0.494	1.27 (0.68–2.36)	0.450
Third trimester	460 (29.10)	1.46 (1.04–2.06)	0.030	1.42 (1.01–2.03)	0.050	1.16 (0.75–1.80)	0.504	1.09 (0.70–1.69)	0.710
**CD4**^**+**^ **category, cells/mm**^**3**^	**1581**								
0–100	37 (2.34)	Ref	-	Ref	-	Ref	-	Ref	
101–200	119 (7.53)	0.39 (0.14–1.12)	0.081	0.38 (0.13–1.10)	0.074	2.02 (0.46–8.91)	0.352	2.01 (0.44–9.21)	0.370
200–350	351 (22.20)	0.55 (0.23–1.34)	0.188	0.53 (0.21–1.29)	0.162	1.16 (0.29–4.63)	0.836	1.11 (0.27–4.61)	0.884
>350	870 (55.03)	0.51 (0.22–1.19)	0.117	0.48 (0.20–1.15)	0.099	1.36 (0.35–5.26)	0.658	1.43 (0.35–5.81)	0.621
Missing	204 (12.90)	0.49 (0.19–1.26)	0.138	0.43 (0.16–1.14)	0.090	1.52 (0.36–6.33)	0.568	1.72 (0.38–7.74)	0.481
**Age, years**	**1581**								
25–34	758 (47.94)	Ref	-	Ref	-	Ref	-	Ref	-
15–24	638 (40.35)	1.21 (0.87–1.70)	0.260	1.22 (0.86–1.72)	0.266	1.28 (0.82–2.0)	0.277	1.30 (0.83–2.03)	0.241
≥35	185 (11.70)	0.97 (0.56–1.66)	0.904	0.92 (0.53–1.60)	0.764	0.93 (0.48–1.81)	0.834	0.90 (0.47–1.72)	0.747
**Antenatal clinic type**	**1576**								
Rural clinic	835 (52.81)	Ref	-	Ref	-	Ref	-	Ref	-
Peri-urban	737 (46.62)	0.80 (0.58–1.11)	0.180	0.77 (0.55–1.08)	0.135	0.57 (0.35–0.93)	0.024	0.59 (0.36–0.97)	0.037
Other	4 (0.25)	2.51 (0.26–24.34)	0.428	2.68 (0.26–27.39)	0.407	-	-	-	-
Missing*	5 (0.32)	-	-	-	-	-	-	-	-
**Calendar year of delivery**	**1581**								
2010	96 (6.07)	Ref	-	Ref	-	Ref	-	Ref	-
2011	342 (21.63)	0.71 (0.37–1.38)	0.311	0.74 (0.37–1.47)	0.388	1.02 (0.44–2.34)	0.970	0.99 (0.42–2.34)	0.975
2012	228 (14.42)	0.35 (0.16–0.79)	0.011	0.38 (0.17–0.87)	0.021	0.80 (0.33–1.98)	0.632	0.79 (0.31–2.01)	0.620
2013	258 (16.32)	0.68 (0.34–1.37)	0.284	0.87 (0.42–1.80)	0.704	0.93 (0.39–2.21)	0.862	1.01 (0.41–2.50)	0.985
2014	399 (25.24)	0.88 (0.46–1.66)	0.688	1.20 (0.60–2.40)	0.616	0.89 (0.39–2.04)	0.783	0.92 (0.37–2.25)	0.850
2015	258 (16.32)	0.68 (0.34–1.37)	0.284	0.95 (0.45–2.01)	0.893	0.68 (0.28–1.70)	0.416	0.71 (0.27–1.89)	0.494

*Dropped category as predicts failure perfectly

Antenatal clinic type, VL, pregnancy count, delivery place and infant sex were excluded from the model as *P* >0.2.

Multivariably, adjusting for ART timing, age, CD4^+^, antenatal clinic and calendar delivery year, NVP-based ART (aOR 1.77; 95% CI 0.89–3.51) and ZDV prophylaxis (aOR 1.03; 95% CI 0.68–1.58) were not associated with PTD versus TDF-(3TC/FTC)-EFV (Table 4).

In univariable analysis, SGA risk for NVP-based ART was 1.74 (95% CI 0.79–3.86) and 1.09 (95% CI 0.72–1.65) for ZDV versus TDF-(3TC/FTC)-EFV. Multivariably, NVP-based ART (aOR 1.55; 95% CI 0.66–3.61) and ZDV prophylaxis (aOR 0.89; 95% CI 0.53–1.47) were not associated with SGA risk versus TDF-(3TC/FTC)-EFV.

In sensitivity analysis (S2 Table), a significantly higher PTD risk for NVP-based ART versus TDF-(3TC/FTC)-EFV was observed (aOR 3.47, 95% CI 1.17–10.31) in the stricter population definition but the confidence intervals were imprecise. When we restricted to seven clinics and calendar year 2012–2015, PTD estimates for NVP-based ART were similar to analysis 1 but lost significance (aOR 3.53; 95% CI 0.50–24.84). PTD and SGA risk for ZDV prophylaxis was not substantially altered when restricting clinics or calendar year. LBW estimates for all regimens were substantially lower than PTD risk in the main analysis with improved precision (analysis 4). When considering lead time bias (analysis 5), PTD risk for NVP-based ART and ZDV mimicked the main analysis; ZDV PTD risk increased from 1.03 in the main analysis to 1.60 (95% CI 0.94–2.71) and SGA risk was the slightly lower than the main analysis with improved precision (aOR 0.76; 95% CI 0.46–1.25).

### Risk factors for PTD and SGA

#### Preconception ART

In adjusted analysis, only calendar delivery year was significantly associated with preconception PTD and SGA. PTD risk declined over calendar time (to 0.13 in 2015) versus 2010 with wide confidence intervals (95% CI 0.02–0.82). SGA risk increased to 4.16 in 2012 versus 2010, but the confidence interval was wide (95% CI 1.14–15.07) (Table 3)

#### Post-conception ARV

Multivariably, third trimester initiations increased PTD risk to 1.42 (95% CI 1.01–2.03) with similar effect size as crude analysis. PTD significantly declined to 0.38 in 2012 versus 2010 (95% CI 0.17–0.87) (Table 4).

Attending peri-urban antenatal clinics versus rural clinics lowered SGA risk to 0.57 (95% CI 0.35–0.93) in univariable analysis with almost no change in effect size in multivariable analysis. There were no other PTD and SGA risk factors

## Discussion

In this study in a high HIV prevalent setting in rural South Africa, 9% of overall deliveries were PTD and 10% were SGA, with minimal overlap between outcomes. PTD births declined over time while SGA births remained relatively stable over six years. There were no significant differences in PTD or SGA risk among women on either preconception or post-conception TDF-(3TC/FTC)-EFV versus other regimens.

In the current study, PTD rates were lower in 2015, most likely reflecting ART advantages for maternal health. In 2010, the national PTD rate was 8.0 per 100 births [38]. We showed a downward PTD trend through 2015 when ART evolved from NVP-based ART and ZDV prophylaxis to TDF-(3TC/FTC)-EFV regimens [31]. The significant PTD risk reduction may reflect global and national commitment to improve newborn health [39–42], including expanded ART access [24]. We also report lower PTD and SGA rates than an earlier Hlabisa study of ART-naïve HIV-infected and -uninfected mothers delivering through 2004, where 21.4% of live born HIV-exposed infants were PTD and 16.6% were SGA [43]. Maternal HIV infection was significantly associated with SGA birth, but not with PTD, consistent with strong evidence linking untreated maternal infection to adverse birth outcomes [44]. The 2010 PTD birth proportion in our study is slightly higher than the Kesho Bora trial (including Hlabisa patients) ending in 2008 where women initiated post-conception ART from 28 weeks; PTD risk was 13% on PI-based ART and 11% in the ZDV prophylaxis group [45].

The reasons behind the sharp variation in 2012 SGA and PTD births are unclear, although several concomitant changes around this time occurred viz: the Hlabisa HIV Programme funding ending in 2012; and changes to the 2013 treatment guidelines to start pregnant women on Option B [24]. Sensitivity analysis reflects these fluctuations as preconception SGA and post-conception PTD estimates became more precise when restricting to 2012–2015 and then excluding calendar year from models. PTD and SGA rates reported in this study are lower than other countries like Botswana; our lower rates may reflect survival bias as we were unable to ascertain neonatal mortality. Moreover, data were collected by routine health staff; women with pregnancy complications or sicker infants with adverse outcomes may have been transferred to referral hospitals with consequent incomplete or missing data on adverse birth outcomes at primary care level. Neonatal morbidity and mortality related to ART are important outcomes for future evaluation.

Preconception, there was no difference in PTD when taking TDF-(3TC/FTC)-EFV versus other regimens in this study. While the adjusted SGA risk was higher in women on NVP-based regimens versus TDF-(3TC/FTC)-EFV, the difference was not significant. In the Botswana surveillance cohort, all preconception regimens (including TDF-FTC-NVP and ZDV-3TC-NVP) had a significantly higher adjusted PTD and SGA risk versus preconception TDF-FTC-EFV [21].

Post-conception, there was a higher non-significant PTD and SGA risk in the NVP-based group versus TDF-(3TC/FTC)-EFV in this study. There was no difference in PTD and non-significant lower risk of SGA births when exposed to ZDV versus TDF-(3TC/FTC)-EFV in this study. Our findings are consistent with Botswana where there was no difference in PTD in women initiating TDF-FTC-EFV versus other 3-drug ART or ZDV; SGA births were less common in women initiating TDF-FTC-EFV versus any other 3-drug ART (aOR 0.4; 95 CI 0.2–0.7) [20]. In our sensitivity analysis (post-conception ARV) restricted to seven clinics with data for 2010 to 2015, NVP-based ART had significantly higher PTD risk versus TDF-(3TC/FTC)-EFV. Since PTD risk was similar when assessing the seven clinics from 2012–2015 and decreased when calendar year was excluded, it is plausible that the lower TDF-(3TC/FTC)-EFV event rate in this study reflects unspecified confounding and information bias with less preterm data collected in the TDF-(3TC/FTC)-EFV group before TDF-FTC-EFV implementation in 2013.

In terms of other risk factors, only third trimester initiation increased PTD risk versus second trimester in the post-conception ARV group in this study. This contrasts to an earlier South African cohort where early pregnancy ART with NNRTI-based ART increased PTD [46]. These dissimilar results in our study are likely related to residual confounding, measurement error or selection bias from late third trimester antenatal attendance care with less time to prevent pregnancy complications and adverse outcomes.

Published results are difficult to synthesize and interpret due to methodological variation, including disparate study designs (e.g. clinical trial, prospective cohort, retrospective cohort, surveillance) and analytic approaches, different populations, settings and obstetric care standards and differing regimens, reflecting time period as well as place. For instance, the Botswana surveillance cohort included 45% of the country’s birth data and reported higher PTD and SGA risk overall. Our cohort includes birth outcomes from a district level hospital and primary health care facilities and excluded stillborn deliveries. Moreover, as none of the Botswana participants received TDF-3TC-EFV or non-TDF EFV-based regimens [20,21], predominant South African first line regimens, applicability to the South African context, especially the preconception group may be limited.

Study strengths include the large pregnancy cohort on predominantly EFV-based ART in a developing setting. Women who initiated post-conception ARV may be different from women on ART for their own health; we thus conducted separate analyses for these groups. This study is generalizable to other resource-limited settings in South Africa.

The main study limitation was heterogeneity between the compared population in both the pre- and post-conception models. Consequently, ART groups were small, especially NVP-based ART and preconception other 3-drug EFV-based regimens, resulting in the study not being sufficiently powered to detect differences with TDF-(3TC/FTC)-EFV. As there were no differences in birth outcomes between TDF-FTC-EFV and TDF-3TC-EFV (or NVP-based regimens) in crude analyses, it is unlikely that combining groups diluted results. We lacked data on some specific known risk factors for PTD and SGA such as hypertension and other comorbidities, maternal weight and nutrition and substance use and cannot rule out residual confounding. In a prior KwaZulu-Natal study, pregnancy smoking, alcohol and illicit drug use were under 10% [47]. We were unable to validate delivery gestational age which was based on LMP and physical examination. In sensitivity analysis, LBW outcomes were slightly different from the PTD post-conception group and vastly different from the preconception group, suggesting more issues around gestational dating in women starting preconception ART. Nonetheless, LMP for gestational age assessment is common in South Africa and may be a reliable gestational age measurement in resource-limited settings [48,49]. PTD risk did not alter substantially when ART timing was more strictly defined in the post-conception sensitivity analysis suggesting lead time bias is unlikely. We did not include an HIV-uninfected group which limits our ability to interpret time-related changes from ART versus health system factors.

## Conclusions

Preconception TDF-(3TC/FTC)-EFV and post-conception TDF-(3TC/FTC)-EFV was not associated with PTD and SGA versus other ART. As there was limited power to detect differences between ART groups, the results should be interpreted with caution. Larger studies are required to elucidate ART regimens, timing effect, and specific factors that determine safe birth outcomes in ART-exposed HIV-infected women in this setting.

## Supporting information

S1 Table Main and sensitivity analyses for estimated effect of preconception ART on PTD and SGA birth outcomes among HIV-infected women in South Africa, 2010–2015. (DOCX)

S2 Table Main and sensitivity analyses for estimated effect of post-conception ART on PTD and SGA birth outcomes among HIV-infected women in South Africa, 2010–2015. (DOCX)
