# Supplementary material for: Preterm delivery and small-for-gestation outcomes in HIV-infected pregnant women on antiretroviral therapy in rural South Africa: Results from a cohort study, 2010-2015
Source: PLoS One. 2018 Feb 22;13(2):e0192805. doi: 10.1371/journal.pone.0192805 (PMC5823389; doi:10.1371/journal.pone.0192805)
Supplement: S1 Table — (DOCX) [file pone.0192805.s001.docx]

**S1 Table. Main and sensitivity analyses for estimated effect of preconception ART on PTD and SGA birth outcomes among HIV-infected women in South Africa, 2010-2015**

|  | **Main analysis (N=968)** | **1. Restricted to seven clinics (N=531)** | **2. Restricted to 2012-2015 calendar delivery year (N=796)** | **3. Excluding calendar delivery year (N=968)** | **4. LBW outcome (N=968)** |
| --- | --- | --- | --- | --- | --- |
| **PTD** | **aOR**  **(95% CI)** | **aOR**  **(95% CI)** | **aOR**  **(95% CI)** | **aOR**  **(95% CI)** | **aOR**  **(95% CI)** |
| TDF-(3TC/FTC)-EFV | Ref | Ref | Ref | Ref | Ref |
| NVP-based regimen | 0.66 (0.27-1.63) | 0.79 (0.21-2.99) | 0.69 (0.23-2.12) | 1.13 (0.51-2.48) | 0.76 (0.28-2.10) |
| Other 3-drug EFV-based regimen | 0.72 (0.24-2.12) | 1.35 (0.29-6.34) | 0.59 (0.13-2.67) | 1.45 (0.59-3.57) | 1.28 (0.37-4.40) |
|  |  |  |  |  |  |
| **SGA** |  |  |  |  |  |
| TDF-(3TC/FTC)-EFV | Ref | Ref | Ref | Ref |  |
| NVP-based regimen | 0.75 (0.40-1.42) | 0.93 (0.38-2.27) | 0.92 (0.49-1.75) | 0.69 (0.39-1.20) |  |
| Other 3-drug EFV-based regimen | 1.55 (0.76-3.16) | 2.22 (0.85-5.79) | 1.43 (0.66-3.11) | 1.27 (0.72-2.23) |  |
