# Supplementary material for: Preterm delivery and small-for-gestation outcomes in HIV-infected pregnant women on antiretroviral therapy in rural South Africa: Results from a cohort study, 2010-2015
Source: PLoS One. 2018 Feb 22;13(2):e0192805. doi: 10.1371/journal.pone.0192805 (PMC5823389; doi:10.1371/journal.pone.0192805)
Supplement: S2 Table — (DOCX) [file pone.0192805.s002.docx]

**S2 Table. Main and sensitivity analyses for estimated effect of post-conception ART on PTD and SGA birth outcomes among HIV-infected women in South Africa, 2010-2015.**

|  | **Main analysis (N=1581)** | **1. Restricted to seven clinics (N=1117)** | **2. Restricted to 2012-2015 calendar delivery year**  **(N=1138)** | **3. Excluding calendar delivery year (N=1576)** | **4. LBW outcome (N=1572)** | **5. Restricted to women starting ART before 24 weeks (N=1275)** |
| --- | --- | --- | --- | --- | --- | --- |
| **PTD** | **aOR**  **(95% CI)** | **aOR**  **(95% CI)** | **aOR**  **(95% CI)** | **aOR**  **(95% CI)** | **aOR**  **(95% CI)** | **aOR**  **(95% CI)** |
| TDF-(3TC/FTC)-EFV | Ref | Ref | Ref | Ref | Ref | Ref |
| NVP-based ART ^b^ | 1.77 (0.89-3.51) | 3.47 (1.17-10.31) | 2.03 (0.58-7.07) | 1.28 (0.68-2.39) | 1.09 (0.43-2.73) | 1.54 (0.56-4.25) |
| ZDV prophylaxis ^a^ | 1.03 (0.68-1.58) | 1.36 (0.75-2.45) | 0.93 (0.55-1.57) | 0.79 (0.55-1.14) | 0.78 (0.44-1.36) | 1.60 (0.94-2.71) |
|  |  |  |  |  |  |  |
| **SGA** |  |  |  |  |  |  |
| TDF-(3TC/FTC)-EFV | Ref | Ref | Ref | Ref |  | Ref |
| NVP-based ART ^b^ | 1.55 (0.66-3.61) | 1.24 (0.37-4.17) | 2.68 (0.48-14.78) | 1.59 (0.71-3.54) |  | 1.29 (0.55-3.03) |
| ZDV prophylaxis ^a^ | 0.89 (0.53-1.47) | 0.60 (0.31-1.16) | 0.75 (0.35-1.63) | 0.93 (0.60-1.43) |  | 0.76 (0.46-1.25) |
